# TransTEx: novel tissue-specificity scoring method for grouping human transcriptome into different expression groups

**DOI:** 10.1093/bioinformatics/btae475

**Published:** 2024-08-09

**Authors:** Pallavi Surana, Pratik Dutta, Ramana V Davuluri

**Affiliations:** Department of Biomedical Informatics, Stony Brook University, Stony Brook, NY 11794, USA; Department of Biomedical Informatics, Stony Brook University, Stony Brook, NY 11794, USA; Department of Biomedical Informatics, Stony Brook University, Stony Brook, NY 11794, USA

## Abstract

**Motivation:**

Although human tissues carry out common molecular processes, gene expression patterns can distinguish different tissues. Traditional informatics methods, primarily at the gene level, overlook the complexity of alternative transcript variants and protein isoforms produced by most genes, changes in which are linked to disease prognosis and drug resistance.

**Results:**

We developed TransTEx (Transcript-level Tissue Expression), a novel tissue-specificity scoring method, for grouping transcripts into four expression groups. TransTEx applies sequential cut-offs to tissue-wise transcript probability estimates, subsampling-based *P*-values and fold-change estimates. Application of TransTEx on GTEx mRNA-seq data divided 199 166 human transcripts into different groups as 17 999 tissue-specific (TSp), 7436 tissue-enhanced, 36 783 widely expressed (Wide), 79 191 lowly expressed (Low), and 57 757 no expression (Null) transcripts. Testis has the most (13 466) TSp isoforms followed by liver (890), brain (701), pituitary (435), and muscle (420). We found that the tissue specificity of alternative transcripts of a gene is predominantly influenced by alternate promoter usage. By overlapping brain-specific transcripts with the cell-type gene-markers in scBrainMap database, we found that 63% of the brain-specific transcripts were enriched in nonneuronal cell types, predominantly astrocytes followed by endothelial cells and oligodendrocytes. In addition, we found 61 brain cell-type marker genes encoding a total of 176 alternative transcripts as brain-specific and 22 alternative transcripts as testis-specific, highlighting the complex TSp and cell-type specific gene regulation and expression at isoform-level. TransTEx can be adopted to the analysis of bulk RNA-seq or scRNA-seq datasets to find tissue- and/or cell-type specific isoform-level gene markers.

**Availability and implementation:**

TransTEx database: https://bmi.cewit.stonybrook.edu/transtexdb/ and the R package is available via GitHub: https://github.com/pallavisurana1/TransTEx.

## 1 Introduction

Gene expression regulation varies across cell types, developmental stages, tissues, populations, and species (Davuluri *et al.* 2008). About ∼50k genes (coding and noncoding) map to ∼200k isoforms implying that genes have two or more protein isoforms that are known to influence gene expression generated by alternative splicing of mRNA (Schneider *et al.* 2017). This means that isoforms are the true expression units. Majority of mRNA expression studies define gene expression as the summation of all the isoform expression values of a gene which are known to be expressed uniquely in a tissue or multiple tissues with diverse expression patterns. Hence, there is a need to explore isoform-level tissue-specific (TSp) expression to understand their diversity and role in regulatory mechanisms during development and disease. In addition, there is a significant association of tissue specificity in disease initiation and progression which, can thereby assist in understanding drug-target interactions, biomarker discovery, prognosis, and diagnosis of complex diseases like cancer (Schneider *et al.* 2017, Tung and Lin 2022).

Existing approaches to determine gene tissue specificity include the TiGER database (Liu *et al.* 2008) for expression enrichment, ROKU specificity using Shannon entropy (Kadota *et al.* 2006), Tissue Specificity Index (Julien *et al.* 2012, Barshir *et al.* 2021) based on expression differences, and the widely accepted τ (Tau)-based scoring (Kryuchkova-Mostacci and Robinson-Rechavi 2017). Additionally, gene network inference, through network analysis (Sonawane *et al.* 2017) or Bayesian methods, models regulatory interactions (Greene *et al.* 2015), aiding in understanding tissue specificity about biological functions and diseases. Subsequently, different studies identified TSp transcripts by applying weighted gene co-expression network analysis (Zhu *et al.* 2016), Shannon entropy (Shi *et al.* 2022), and tau-score (Kryuchkova-Mostacci and Robinson-Rechavi 2017, Lüleci and Yılmaz 2022). TEx-MST database provides normal tissue expression patterns of MANE select transcripts (Tung and Lin 2022).

Many RNA analysis methods do not consider the quality of RNA samples, the true expression values at the isoform level, or the significance of expression changes among different methods. Most of the methods, which rely on mean expression estimates, often yield inconsistent results, with some studies identifying fewer TSp transcripts compared to others. This lack of consistency is attributed to the absence of the gold standard dataset. Grouping of the whole transcriptome into different expression groups hence becomes imperative to comprehend TSp gene regulation as, over 50% of human genes are affected by alternative splicing and transcriptional events (Wang *et al.* 2008).

To this end, we propose Transcript-level Tissue Expression (TransTEx): a scoring method to address these challenges by categorizing transcripts into five expression classes, based on their unique or multiple tissue expression patterns, to understand TSp gene regulation in normal and disease conditions (Thul and Lindskog 2018). The five expression classes are: (i) TSp: the transcripts uniquely expressed in a tissue; (ii) TEn: transcripts expressed in one or more but less than 50% of the tissues; (iii) Wide:transcripts expressed in >50% of tissues; (iv) Low:transcripts that show low expression or expressed in only a small subset of samples of across all tissues; and (v) Null:transcripts with no expression or expressed only in minimal number of samples in all the tissues. The derived TSp transcript set is compared with SRTdb (Shi *et al.* 2022) and τ-score (Kryuchkova-Mostacci and Robinson-Rechavi 2017) based TSp transcripts. In addition, all three TSp transcripts are compared with protein-level TSp genes from Human Protein Atlas (HPA) database (Pontén *et al.* 2008). As a major contribution of this study, we provide a searchable database of the TransTEx transcript grouping along with the tissue-wise expression patterns.

## 2 Materials and methods

### 2.1 Datasets

We downloaded the isoform level mRNA expression data (Transcript per million or TPM expression estimates) consisting of 17 382 samples, corresponding to 838 post-mortem donors across 30 major body tissues, from Genotype-Tissue Expression data portal (GTEx, V8 release) (GTEx Consortium 2015, 2020). The subregions within a tissue, like in brain, were considered as one tissue group for this study. Data for TSp comparisons was downloaded from SRTdb, which includes human tissue and cancer specific transcripts (Shi *et al.* 2022), HPA consortium database and filtered for Tissue-enriched expression type which include both unique expression and elevated expression in tissue groups (Pontén *et al.* 2008).

### 2.2 Description of TransTEx scoring method

We developed TransTEx scoring method to categorize all the human gene transcripts, including those derived by alternative transcription and/or alternative splicing, into five different expression classes—(i) TSp, (ii) TEn, (iii) Wide, (iv) Low, and (5) Null. The steps of TransTEx method are described in the following sections.

#### 2.2.1 Identification of transcripts above baseline expression

A transcript is considered greater than baseline expression in a sample if its TPM ≥ 0.5 (Duffy *et al.* 2020, Moreno *et al.* 2022). We define the probability of *j*^th^ transcript expressed in *i*^th^ tissue as, **P_ij_** = number of samples with TPM_*ij*_ ≥0.5/total number of samples in *i*^th^ tissue. We plotted the distribution of **P_ij_** values to determine all the transcripts that are consistently expressed in most of the samples in each tissue. Next, right most inflection point (**R-IP_i_**) was calculated as a cut-off for *P_ij_* to determine the expressed transcripts in *i*^th^ tissue. Inflection point is a point on the density plot where the direction of the curvature changes (i.e. second derivative is 0). Here R-IP_i_ corresponds to change in the curvature in the probability curve within the range (0.75–0.9). The transcripts with expression probabilities above R-IP_i_ correspond to expressed transcripts that have higher than the minimal expression in majority samples of the *i*^th^ tissue.

#### 2.2.2 Identification of TSp and TEn transcripts

Next, we applied two more cut-offs to determine the transcripts with significantly higher expression in a tissue/group of tissues compared to the rest. They include calculating a novel fold change for transcript expression in the tissue of interest compared to the tissues with the most similar expression and, empirical-p (**EMP-p**) values to assess statistical significance. For the fold change calculation, we first calculate mean expression of *j*^th^ transcript in *i*^th^ tissue (mTPM_*ij*_). Then we find max[mTPM_*kj*_ (*k* ≠ *i*)], maximum mean TPM value of *j*^th^ transcript in the rest of the tissue groups. Hence, **FC-MAX_ij_** = log_2_(mTPM_*ij*_/max[mTPM_*kj*_ (*k* ≠ *i*)]) (Feng *et al.* 2012). To identify transcripts with significantly higher expression in a tissue of interest over the rest of the tissues, we applied stratified subsampling-based simulation method to estimate EMP-p (Bickel *et al.* 2010, Pundir *et al.* 2021) (Supplementary Methods S1).

#### 2.2.3 Grouping of transcripts into different expression classes

Next, we follow the steps below to group a transcript into one of the five expression classes.

1. For a given transcript *j*, find the number of tissues (*k*) in which the probability of its expression is above R-IP_*i*_ cut-off (*P_ij_* ≥ R-IP_*i*_ is true for *i* = 0 to *k*).
2. Then, the *j*th transcript is grouped as:
  - Low or no expression (Low transcripts) if *k* = 0.
  - TSp expression (TSp transcripts) if *k* = 1.
  - Tissue-enhanced expression (TEn transcripts) if *k* = 2 to 50% of tissues (13 tissues).
  - Widespread expression (Wide transcripts) if *k* > 50% of tissues (14–26 tissue groups).
3. For the TSp and TEn transcripts, we calculate FC-MAX_*ij*_ and EMP-p values. Next, group those that have both FC-MAX_*ij*_≥1 and EMP-p≤0.05 into TSp or TEn expression classes.
4. For *k* = 0 transcripts, group those in Null expressed group if *P_ij_* < L-IP_i_ (left peak cutoff) is true for all tissues. Otherwise, group those transcripts in Low expression group.

#### 2.2.4 Comparison with existing methods

Most tissue specificity calculations suggest the usage of τ-based scoring followed by Gini index (Kryuchkova-Mostacci and Robinson-Rechavi 2017, Jiang and Chen 2022) as it outperforms other methods like *z*-score, tissue specificity index, counts and expression enrichment (Supplementary Methods S2). Therefore, we calculate τ-score to find TSp transcripts and compare this with TransTEx in addition to SRTdb’s Shannon entropy measure (Supplementary Methods S3). Next, TSp transcripts from TransTEx, τ-score, and SRTdb are compared with experimentally validated genes in the HPA (Supplementary Methods S4).

### 2.3 Transcriptional and splicing events analysis

The TSp transcripts expression class is divided into single-transcript and multitranscript genes. For every G gene that has *j_n_*_= 1_ transcripts, we have single-transcript genes. Now, for the remaining multitranscript genes *j_n_*_> 1_ where *n* > 1 transcript belongs to a single gene. For each *G*, *j_n_* has information about transcription start sites (TSS), exon and intron coordinates. We classify the multitranscript genes into single and multipromoter groups based on their TSS locations. A single-promoter gene G has the same TSS position within a threshold (TSS_*j*__1_ < TSS_*t*_, where ∑(*j* = 1 to *n*)), and multipromoter gene G has different TSS positions (TSS_*j*__1_ > TSS_*t*_). TSS_*t*_ is a threshold, above which we group transcripts as multitranscript genes, which has values 50, 500, and 5000 (Sammeth, Foissac and Guigó 2008). R packages BioMart and Genomic Ranges are used to fetch all the information about transcripts, genes, and their positions (Durinck *et al.* 2005, Lawrence *et al.* 2013).

### 2.4 Enriched cell types and pathway analysis

To investigate the pathways associated with brain-specific transcripts, we use mapped gene IDs to find related pathways. We report GO (Gene Ontology), DisGeNET, and KEGG pathways, from the R package ‘clusterProfiler’ (Wu *et al.* 2021). The scBrainmap database which has cell-type gene markers (only human species used in this study) in age groups ranging from weeks to years’ post-birth excluding stages post-fertilization was used to compare with brain-specific mapped genes (Chi *et al.* 2023). This aligns with our use of data from GTEx, which predominantly consists of post-mortem samples. We also look at the cell types enriched using a web based tool with other tissues like lung, heart, pituitary, liver, and muscle using WebCSEA (Dai *et al.* 2022). This generates a web-based cell-type specific enrichment analysis across 1355 human tissue cell types. This comparative analysis was done at the gene level due to the limited availability of isoform-level data.

## 3 Results

### 3.1 TransTEx classifies transcripts into five expression classes

We developed TransTEx method to accurately determine TSp and TEn transcripts that are consistently expressed above baseline in majority of the tissue samples. Applying this method to the transcript-level expression profiles in 10 076 samples across 26 major tissue groups in GTEx, we grouped 199 166 transcripts into four expression classes. We discarded samples with RNA integrity number (RIN) < 7 and tissues with < 50 samples (Supplementary Table S1). RIN is a standardized score that measures the integrity of RNA samples for sequencing applications ranging from 10 (highly intact RNA) to 1 (completely degraded RNA).

**Figure 1.**
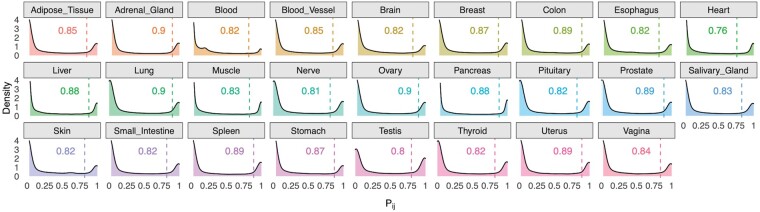
Distributions of the tissue-wise transcript expression probabilities (*P_ij_*). The density plots resemble U-shaped beta-distribution with two peaks at extreme ends. For each tissue, inflection point (R-IP_*i*_) is computed by considering *P_ij_* > 0.5 as the right peak of the distribution represents success (expression of transcripts in a tissue).

By plotting the transcript probabilities (*P_ij_*’s) in each tissue (Fig. 1), we observed U-shaped beta-distribution. While the left peak of the beta distribution corresponds to transcripts with low expression probability (*P_ij_* < 0.25), those in the right peak (*P_ij_* > 0.75) correspond high expression probability or success of *j*^th^ transcript expression in the *i*^th^ tissue. We calculated R-IP_*i*_ as a first threshold to determine the transcripts that are expressed in majority of the samples in the *i*^th^ tissue. We found that while 91 742 (46%) transcripts corresponding to 27 592 genes (both protein-coding and noncoding) satisfied the condition *P_ij_* ≥ R-IP_*i*_ in one or more tissues, 25 566 transcripts (13%) corresponding to 16 507 genes satisfied *P_ij_* ≥R-IP_*i*_ in only one tissue (Table 1, Supplementary Table S2). We found 17 665 TSp transcripts (10 635 genes), with significantly higher mean expression than the nearest tissue by applying cut-offs for EMP-p ≤ 0.05 and FC-MAX_*ij*_ ≥ 1. Based on Ensembl 110 release, 320 transcripts have been deprecated (GTeX v8, last updated in September 2020 built on annotations from Ensembl v101). Further, we found that 457 transcripts belong to Low expression group across all tissues with L-IP_*i*_ < *P_ij_* ≤ R-IP_*i*_.

**Table 1.** Summary of the transcripts (genes) that pass different cut-offs of TransTEx algorithm.^a^

Tissue	R-IP_i_ cut-off	No. of transcripts with		
		*P_ij_* ≥ R-IP_*i*_	*P_ij_* ≥ R-IP_*i*_ (unique in *i*th tissue)	(*P_ij_* > R-IP_*i*_) and log 2(FC-MAX_*ij*_) ≥ 1 and EMP-p ≤ .05
Adipose tissue	0.78	41 260 (14 544)	69 (60)	2 (2)
Adrenal gland	0.9	34 158 (13 604)	168 (121)	127 (85)
Blood	0.75	18 630 (8552)	182 (149)	99 (79)
Blood vessel	0.76	41 823 (14 520)	523 (418)	149 (107)
Brain	0.76	36 089 (15 029)	981 (723)	701 (481)
Breast	0.75	45 328 (15 355)	69 (67)	1 (1)
Colon	0.89	32 300 (13 111)	2 (1)	0 (0)
Esophagus	0.75	39 302 (14 077)	17 (16)	1 (1)
Heart	0.76	30 791 (12 851)	270 (193)	159 (100)
Liver	0.75	31 914 (12 741)	1150 (629)	890 (448)
Lung	0.78	47 219 (15 969)	420 (322)	162 (110)
Muscle	0.83	25 727 (11 308)	557 (388)	420 (279)
Nerve	0.81	48 697 (16 262)	775 (622)	284 (199)
Ovary	0.9	38 263 (14 205)	148 (113)	81 (58)
Pancreas	0.88	26 079 (12 013)	262 (142)	200 (98)
Pituitary	0.82	47 598 (16 848)	1043 (786)	435 (317)
Prostate	0.89	39 803 (15 106)	73 (48)	46 (23)
Salivary gland	0.79	43 255 (15 404)	259 (207)	87 (60)
Skin	0.82	34 333 (12 894)	41 (39)	15 (14)
Small intestine	0.78	43 417 (15 558)	222 (186)	62 (51)
Spleen	0.89	40 446 (15 201)	678 (500)	204 (156)
Stomach	0.87	30 508 (12 771)	3 (2)	2 (2)
Testis	0.76	66 362 (23 156)	16 355 (9804)	13 466 (8118)
Thyroid	0.77	50 591 (16 680)	752 (558)	294 (177)
Uterus	0.89	39 845 (14 643)	103 (76)	52 (32)
Vagina	0.76	45 999 (15 677)	444 (337)	60 (41)
Total		91 742 (27 592)	25 566 (16 507)	17 999 (10 635)

a 334 not found in latest Ensembl V110. Column 2 lists the R-IP_*i*_ values, the right-most IP on the density plot (Fig. 1), where the direction of the curvature changes. Column 3 shows the number of transcripts (genes) with expression probability greater than R-IP_*i*_. While Column 4 presents the number of transcripts that pass the R-IP_*i*_ cut-off uniquely in that tissue, Column 5 gives the number of TSp transcripts in each tissue (above all the three thresholds). Testis has the highest number of TSp transcripts, followed by liver, brain, pituitary, and muscle.

**Figure 2.**
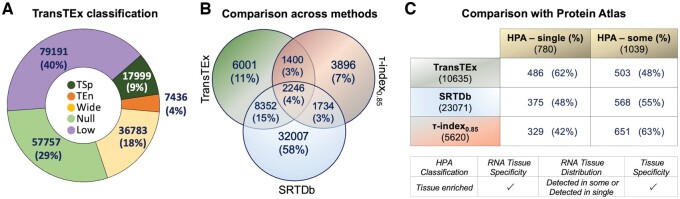
Summary of TransTEx classification and comparison statistics with other methods. (A) The overall distribution of expression classes according to our scoring method (% indicates the number of transcripts in expression class in comparison to the total number of transcripts assessed). (B) Venn diagram showing the overlap of TransTEx with SRTDb and τ-score (0.85 threshold). (C) Overlap of TSp genes with HPA genes distributed in a single tissue or some tissues, defined as tissue enriched in HPA.

TransTEx method categorized 133 242 (67%) transcripts as Null transcripts (50 319 genes). In addition, 36 783 (18%) transcripts were classified as Wide transcripts (13 899 genes) and 7436 (4%) as TEn transcripts (4565 genes) (Fig. 2A). Testis has the highest number of TSp transcripts (13 466), followed by liver (890), brain (701), pituitary (435), and muscle (420) (Table 1). Our analysis found lower than 15 TSp transcripts in adipose, colon, breast, stomach, and esophagus tissues. For genes in a specific expression group, by counting the number of alternative transcripts that fall into the other expression groups, we found that the alternative transcripts were distributed across all the expression groups. For example, while 50% of the total transcripts of genes in Low expression group fall in the Low expression group, the rest 20%, 4%, 7%, and 20% fall in the Null, TEn, TSp, and Wide expression groups, respectively. Similarly, while 26% of alternative transcripts of TSp gene are TSp, the rest are distributed as 36% in Low, 19% in Null, 4% in TEn, and 33% in Wide expression groups (Supplementary Table S3).

### 3.2 Comparison of tissue specificity across methods

Among the 44 339 TSp transcripts from SRTdb, 9856 transcripts intersect with TransTEx (Fig. 2B). By calculating τ-score at 0.85 threshold, we found 9276 TSp transcripts. While STRdb has categorized the highest number of TSp transcripts (44 339), τ-score found the least number of transcripts (9276). Surprisingly, only 2246 transcripts (4%) are categorized as TSp in consensus by all three methods. The overlap between TransTex and STRdb is higher (19%) than the overlap between TransTEx and τ-score (∼7%).

Delving deeper into the significant numbers of inconsistent transcripts across methods, we checked if they match with any non-TSp categories of TransTEx. Notably, 29 132 TSp transcripts from SRTdb are classified into TransTEx’s Null category (Supplementary Table S4) (Pertea *et al.* 2015). We also looked at the TPM expression distribution and compared expression differences for genes that do not agree (Supplementary Fig. S2).

Next, we compared the genes mapped to the TSp transcripts with HPA’s tissue-enriched genes, reported enriched in at least one tissue, categorized as HPA-single tissue (780 genes) and HPA-some tissue distribution (1039 genes). Notably, 62% of HPA-single and 52% of HPA-some genes overlap with TransTEx (Fig. 2C). We found that the highest number of HPA TSp genes (62%) are categorized as TSp genes in TransTEx, followed by SRTdb (48%) and τ-score (42%). Further, the least number of HPA tissue-enriched proteins (enriched in more than one tissue) are categorized as TSp in TransTEx (52%), followed by SRTdb (55%) and τ-score (63%) (Supplementary Table S5). This discrepancy can be attributed to TSp isoforms enriched at the proteome level (HPA) but not at the transcriptome level (TransTEx). Interestingly, other transcripts of genes in the TSp category, are either Low or Wide expression classes according to TransTEx (Supplementary Table S6). This suggests that only some TSp genes at the transcript level are highly expressed and might be close expression patterns in >1 tissue while some others do not even exceed the *P_ij_* threshold.

Further, to validate if Wide transcripts have housekeeping functions, we compared the Wide genes to the database HRT Atlas v1 (Hounkpe *et al.* 2021) which has 2045 unique housekeeping genes. Out of the 36 783 Wide transcripts (Ensembl v110), 684 transcripts do not map to relevant Gene IDs. Among the remaining housekeeping transcripts of HRT atlas, ∼98% (1999) match with TransTEx. This implies that some of the genes constituted in the Wide transcripts have housekeeping functions.

### 3.3 Transcript lengths and biotypes of expression classes

**Figure 3.**
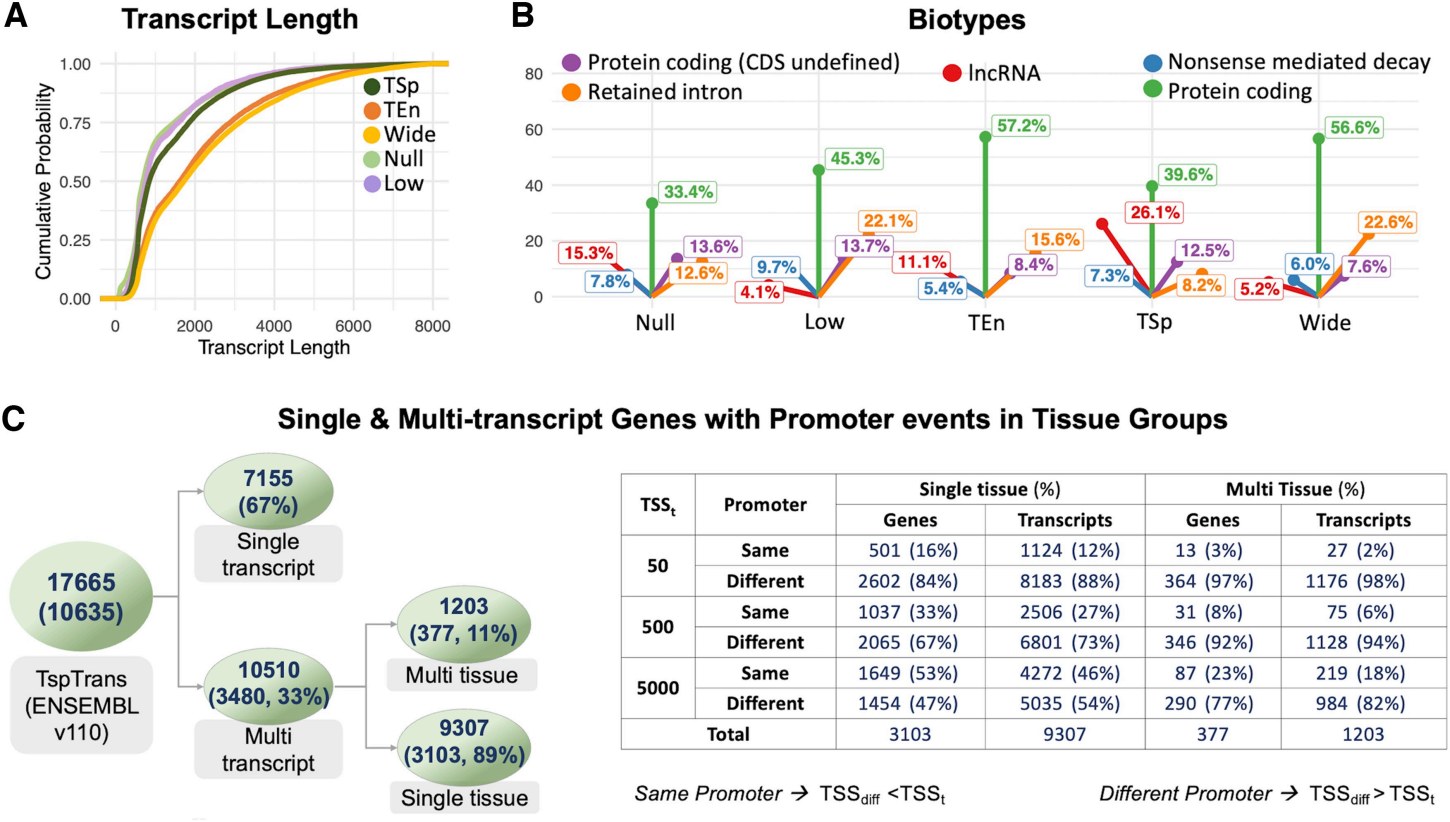
Summary of TransTEx classification and comparison statistics with other methods. (A) Summarizes the transcript length variation across the four different expression classes. (B) Shows the top biological features of transcripts which can indicate their role in human health and development (C) Left—Classification of TransTEx into single transcript and multitranscript genes where the numbers are: number of TSp transcripts (number of mapped genes, % of these genes). Further, they are classified into single-tissue same-gene TSp transcripts or same-gene and multitissue TSp transcripts. Right—Classification of transcriptional and splicing events in multi transcript TSp genes. Summarizes the group in which one gene has transcripts that are tissue specific in different tissues or in the same tissue. *Note*: The classification of promoter events is based on TSS threshold (TSS_t_) of 50, 500, and 5000 bp where TSS difference (TSS_diff_) should be greater than or lesser than TSS_t_ to classify them as the same or different promoter events.

TSp and Wide genes demonstrate distinct transcriptomic characteristics that reflect their unique roles in cellular biology. Transcripts in TSp, Null, and Low expression groups have similar length distributions as compared to Wide and TEn groups. While 50% of the TSp, Null, and Low transcripts are shorter than 1000 bp, only 25% of the TEn and Wide transcripts are shorter than 1000 bp. Likewise, 25% of the TSp, Null, and Low transcripts are longer than 2000 bp, 50% of the TEn and Wide transcripts are longer than 2000 bp (Fig. 3A). By integrating with the ENSEMBL transcript Biotype classification, we found that the majority (nearly 65%) of TEn and Wide transcripts are protein-coding, followed by retained intron category. Among TSp transcripts, 50% are protein coding, followed by lncRNAs (26%). Among the Null expression class, 45% are protein-coding followed by lncRNAs (15%) (Fig. 3B). Collectively, the five expression classes differ in transcript lengths and biotype gene categories.

### 3.4 Alternative transcription underpins tissue specificity

Next, we classified TransTEx’s 10 635 TSp genes into single and multitranscript genes (Fig. 3C, left). We found majority of the genes (67%) have only one TSp transcript per gene, whereas the rest 33% of the genes consist of two or more TSp transcripts per gene. Of the multitranscript Tsp genes, a majority of the alternative Tsp Transcripts (89%) were found to be specific to the same tissue. Alternative TSp transcripts of the remaining (11%) multitranscript genes displayed TSp patterns in different tissues (multitissue) category.

We then, checked whether the alternative TSp transcripts that belong to the same gene are driven by alternative promoters or share the same promoter (Fig. 3C, right). We found that a significantly higher number of multitranscript TSp genes in the multitissue category use alternative promoters. For example, the distance between TSSs of alternative transcripts of 77% of the 377 multitranscript TSp genes (in the multitissue category) is more than 5000 bp, indicating the use of different proximal and regulatory regions. On the contrary, in the single-tissue category, distance between TSS of alternative transcripts of the majority of the multitranscript genes (47%) is less than 5000 bp, indicating the use of same proximal and regulatory promoter in driving the expression of corresponding TSp transcripts (Supplementary Fig. S3) in the same tissue.

Amongst multitranscript TSp genes, the gene with most TSp transcripts is *PNLIPRP1* (19 pancreas) and *MIR9-1HG* (16 brain, 3 testis). Top 10 genes are summarized in Supplementary Table S7A and B. *PNLIPRP1* is pancreatic lipase related protein that is important in lipid metabolic process and low levels of this gene has been implicated in pancreatic cancer (Zhang *et al.* 2013). *MIR9-1HG* is *MIR9-1* host gene is a long noncoding RNA (lncRNA) involved in the positive regulation of RNA-polymerase II which thereby increases transcription rate (Barshir *et al.* 2021). We found that while 1672 genes have two different TSp transcripts, 1431 genes possess three or more different TSp transcripts, demonstrating the TSp expression at isoform-level (Supplementary Table S7C).

### 3.5 Inferences of brain specificity

**Figure 4.**
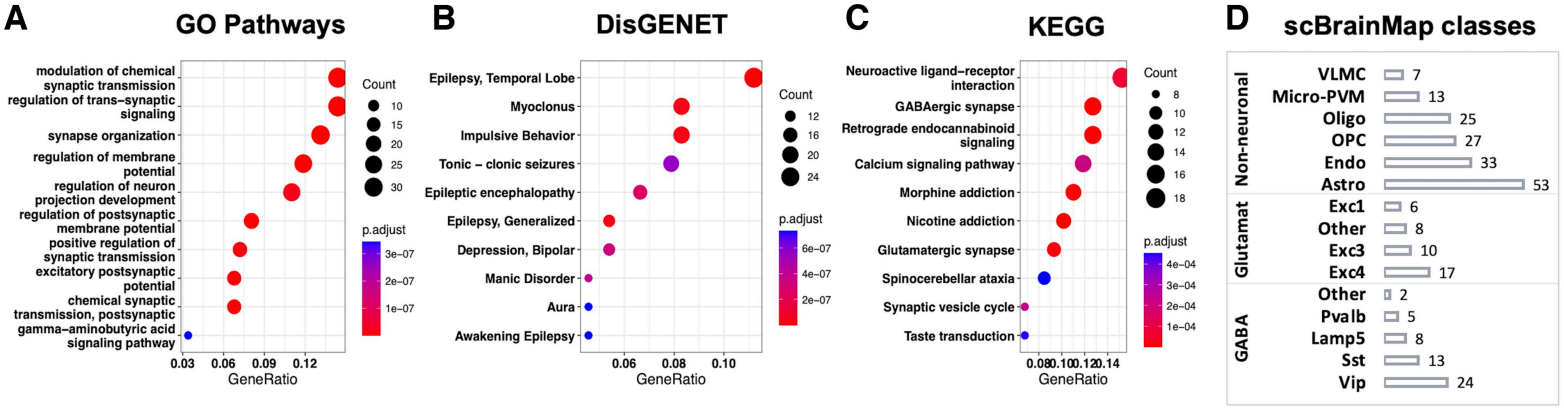
Summarize the pathways enriched in brain TSp transcripts. This is done for genes which map to these transcripts, and it is queried in the (A) GO: biological pathways. (B) DisGENET which has disease-related pathways among these gene lists and (C) KEGG pathways enriched in the gene lists. (D) Number of TSp mapped genes which overlap with the scBrainMap database across developmental stages.

It is known that brain-specific genes are closely tied to neuronal communication and help in the maintenance of neural balance, which is vital for the regulation of synapses and the effect of neurotransmitters (Wu *et al.* 2021). Pathway analysis found that brain-specific genes of TSp transcripts are enriched in key biological pathways, such as GABAergic and Glutamatergic synapses, calcium signaling, and endocannabinoid signaling, as expected (Fig. 4A–C).

We then investigated whether the TSp transcripts are predominantly expressed in a single cell type by performing overlap analysis with the brain cell-type gene markers curated in the scBrainMap database genes (Chi *et al.* 2023). We found that 251 out of 436 Brain-specific transcript genes were annotated as cell-type markers in scBrainMap (human only) (Fig. 4D). While the majority (158 or 63%) of these genes are annotated as nonneuronal cell-type markers, the rest belong to GABAergic (21%) and Glutamatergic (16%) cell-type markers. Within the nonneuronal cell-type markers, astrocytes were the most in number, followed by endothelial and oligodendrocyte precursor cells.

Among the 251 genes, which are both brain specific (based on TransTEx classification) and cell-type marker genes (based on scBrainMap database), 190 genes consist of one TSp transcript per gene, whereas the rest 61 genes have multiple TSp transcripts, with a majority of 176 transcripts in brain and 22 transcripts in testis, highlighting the complex gene regulation and expression at the isoform-level (Supplementary Table S8).

We then extended TransTEx to brain subregions alone and found that none of the transcripts specific across the subregions of the brain are brain-specific (where brain is considered as one tissue without subregions) (Supplementary Table S9A and B). This suggests that brain subregion-specific transcripts differ and help in understanding specificity relevant to a disorder where a subregion of the brain is affected. We found most of these transcripts are enriched in Low, followed by Wide and TSp. Among the subregions, the spinal cord in the cervical C1 vertebra (60% of the transcripts) is enriched the most across other TSp sites. This further highlights the complex tapestry of brain regulation in humans and the need to study brain subregions and their isoform-level expression patterns to better understand complex neurological disorders.

### 3.6 Cell-type specificity of TSp genes

Since brain cell-type marker genes were found to have alternative transcripts as brain-specific and testis-specific, we investigated whether this is common in other tissues as well. By mapping TSp genes in TransTEx to cell-type marker genes curated in WebSCEA (Dai *et al.* 2022), we found 393 (out of 408), 254 (out of 274), 84 (out of 90), 262 (out of 267), and 5663 (out of 6479) as marker genes in liver, pituitary, heart, muscle, and testis, respectively. By focusing on the cell types of four of these (heart, muscle, liver, and testis), we found that these marker genes are enriched in the expected cell types among the top 20 in the jitter plots (Supplementary Fig. S4). It is interesting to observe that ciliated cells of lung, myeloid progenitors, hematopoietic stem cells, and plasma cells are enriched among testis TSp mapped genes among top 20 cell types. This finding is consistent with the cross-tissue specificity explained in Section 3.5 highlighting the relevance of studying the individual isoforms to understand cell type specificity. Next, we compared TSp genes of tissues mentioned in the context of other alternative sites used (if any). We found that the second most dominant site of TSp genes apart from the tissue itself is testis and for testis it is brain (Supplementary Table S10).

## 4 Discussion

Tissue specificity plays a pivotal role in cellular functioning within organisms, influenced by a myriad of factors ranging from transcriptional modifications to splicing changes. Here, we developed TransTEx to classify transcripts, based on mRNA expression in addition to accounting for sample quality (RIN). TransTEx finds that only 9% of the total transcripts (coding and noncoding) in the human genome are TSp of which testis accounts for 75% of the TSp transcripts, also shown in previous studies (Kryuchkova-Mostacci and Robinson-Rechavi 2017, Shi *et al.* 2022). This can be attributed to the role of testis in meiosis through complex processes like chromosomal reduction and nuclear condensation. Sperm cells develop specialized features for motility and fertilization demanding a higher number of transcriptional activities hence required (Djureinovic *et al.* 2014, Zhu *et al.* 2016).

We observed that 67% of the genes mapped to TSp transcripts have a single transcript specificity, while the rest can be single-tissue or multitissue specific. This indicates that multiple isoforms of a gene are not necessary in TSp functions hence, specialized roles in a tissue (Gonzàlez-Porta *et al.* 2013, Jiang *et al.* 2016, de Goede *et al.* 2021). Notably, TSp transcripts include a significant proportion of lncRNA (26%) when compared to other expression classes like Wide transcripts (5%). Hence, the prevalence of lncRNAs increases in classes with expression in fewer tissues as compared to protein coding genes which show a reverse trend. Also, TSp, Null and Low transcripts typically have similar lengths, with half being shorter than 1000 bps, whereas the TEn and Wide categories have more that exceed 2000 bps. This is deemed significant as Wide genes have some housekeeping functions as well which regulate basic cellular processes expressed across multiple tissues (Jiang *et al.* 2016, Kern *et al.* 2018). Studies have found that lncRNAs are more TSp than mRNAs and can be translated into proteins even at low expression levels. However, only a few lncRNAs are translated into proteins and they are generally shorter than protein-coding genes. This could explain why there are few experimentally validated lncRNAs at the protein level (HPA) (Kern *et al.* 2018).

In the alternate promoter analysis, we found that only a few genes express tissue-specificity across multiple tissues. For genes with single tissue specificity, most differences in TSS are <5000 bp, unlike those genes expressed in the multiple tissues, which have greater differences in TSS regions. This suggests that “tissue preference” more accurately describes promoter activity than tissue specificity among multitranscript multitissue-specific genes and the usage of different proximal and regulatory regions (Jacox *et al.* 2010, Pal *et al.* 2011).

These observations provide additional insights into the cell types expressing brain-specific transcripts. Although alternate promoter activity shows a similar pattern, it is noteworthy that nonneuronal cell types such as astrocytes, endothelial cells, and oligodendrocytes, along with pathways linked to neurodegenerative disorders, are prevalent. This suggests that brain-specific transcripts are dysregulated in neurodegenerative disorders and could serve as valuable resources for biomarkers and downstream target validation (Dezso *et al.* 2008). Studies have reported that a higher proportion of isoform switches are associated with the events explained earlier in complex neurodegenerative diseases like Alzheimer’s and cancer. This highlights the crucial roles of both neuronal and nonneuronal cells, including astrocytes and oligodendrocytes, in maintaining brain functions and protective barriers (McKenzie *et al.* 2018, Jurga *et al.* 2021). Overall, we observe high cell-type specificity among the mapped TSp genes in heart, lung, and liver but not in testis. It, hence, becomes integral to study cell type specificity among testis-specific transcripts instead of the genes they belong to.

Though we have developed a robust method for transcriptome grouping into different expression groups, one of the major limitations is the lack of gold standard dataset of TSp genes for comparative analysis (Dezso *et al.* 2008, Zhu *et al.* 2016, Kryuchkova-Mostacci and Robinson-Rechavi 2017, Lüleci and Yılmaz 2022). A recent benchmarking study compared nine different metrics, including tau-score, *z*-score, counts and Gini index reported that most methods were strongly skewed towards classifying majority of the genes as ubiquitous, and fewer as TSp or of intermediate expression groups (Kryuchkova-Mostacci and Robinson-Rechavi 2017). While most of the tissue specificity methods incorporate different statistical properties, such as mean and standard deviation, of the expression estimates, our proposed method determines the transcripts that are consistently expressed (*P_ij_* above baseline) in most of the samples of a tissue. Since, *P_ij_* values followed U-shaped beta-distribution in each tissue, the left and right inflection points of steep upward slope on both ends of the distribution determine transcripts with low and high probability of expression in that tissue. Furthermore, previous studies have reported poor correlation between transcriptome and protein abundance, indicating mRNA levels are not always good predictors for protein abundance (Upadhya and Ryan 2022). Therefore, instead of relying on mRNA abundance estimates, TransTEx method incorporates simple probabilities estimates (expressed or not) by taking advantage of high number of samples in GTEx. Nevertheless, overlap analyses with HPA showed that TSp genes in TransTEx has the highest overlap with the HPA single tissue genes.

In summary, TransTEx method provides a comprehensive classification of human transcriptome into five different categories. Integrative analysis of the TSp transcripts highlights the importance of gene regulation and cell-type marker analyses at splice-variant or at the isoform level. A key finding is that the testis exhibits the highest number of TSp transcripts, followed by liver, brain, pituitary, and muscle. Further, single tissue specificity is the primary characteristic of tissue specificity, while multitissue specificity is driven by alternate promoter usage. On studying brain subregions separately, we found that different set of transcripts are specific and enriched in Low, followed by Wide and Tsp in other tissues. This is a resource to study brain subregion specific disorders and understand their dysregulation in disease. TransTEx could be expanded to include additional mRNA expression datasets for transcript classification based on expression levels. Ultimately, our findings suggest that understanding tissue specificity is crucial for advancing our knowledge of development and disease and can aid in biomarker discovery and implementation. We anticipate that the TransTEx method can be applied to analyze bulk RNA-seq or single-cell RNA-seq datasets to identify tissue- and/or cell-type-specific gene markers at the isoform level.

## Supplementary Material

btae475_Supplementary_Data
