# Supplementary material for: TransTEx: novel tissue-specificity scoring method for grouping human transcriptome into different expression groups
Source: Bioinformatics. 2024 Aug 9;40(8):btae475. doi: 10.1093/bioinformatics/btae475 (PMC11319638; doi:10.1093/bioinformatics/btae475)
Supplement: btae475_Supplementary_Data [file btae475_supplementary_data.zip › TransTex_SI.docx]

**Supplementary Methods**

**1. Stratified subsampling Procedure:**

The following procedure is applied to calculate EMP-p values to find significantly upregulated transcripts in each tissue in comparison to the rest of the tissues (Pundir et al., 2021):

1. Assume all the samples in i-th tissue as Sample-1 and the rest of the samples in 25 tissues together as Sample-2. Consider 25 tissues as different strata in Sample-2. Let the *l*^th^ stratum have *n_l_*, *l*=1,2, …, 25, number of samples, such that $N=\sum_{l=1}^{25} n_{l}$ .
2. Subsampling:
   1. Draw a subsample of size *b_l_* from sample of size *n_l_* from *l*^th^ stratum using simple-random sampling without replacement (SRSWOR).
   2. While sub-sample of size *b_i_* drawn from i-th tissue constitutes SubSample-1, all the subsampling units drawn from the 29 strata (tissue groups) together constitute a stratified subsample of size $b=\sum_{l=1}^{k} b_{l}$, (l≠i), denoted as SubSample-2.
3. Apply one-tailed two sample Wilcoxon signed rank test to compute the test statistic W, for comparing expression of j-th transcript between i-th tissue (Sample-1) and the other tissues (Sample-2).
4. Calculate Emp-p using Monte Carlo simulation procedure: Generate 100 subsamples from the original samples (using procedure in step-2), calculate the test statistic, W_l_, for each pair of the subsamples (l=1,..100) and compare with the test statistic of the overall sample (W_o_). EMP-p value is then calculated as EMP-p = (𝑟+1)/ (100+1), where *r* is the total number of subsamples that result in W_l_, > W_o_ (Bickel et al., 2010).

Stratified subsampling procedure was chosen as it outperforms random subsampling and is not too stringent in estimation of significance (Bickel et al., 2010; Pundir et al., 2021)

**2. τ -based scoring method:**

1. τ-index is calculated as $\sum_{i=1}^{N} \frac{1-xi}{N-1}$ where N is total number of tissues and xi is log2 normalized *mTPM_ij_* expression values from pre-filtered GTEx v8 isoform expression data (same as TransTEx calculation) (Kryuchkova-Mostacci & Robinson-Rechavi, 2017).
2. IP_all_ was found at 0.8 and most studies (Moreno et al., 2022; Duffy et al., 2020) use 0.85 as shown **Supplementary Figure 1***.* Hence, we choose IP_i_ or threshold as 0.85*.*
3. Next, we define three expression groups here based on the τ values which range from 0 to 1, Low expression (estimates for i-th tissue < 1 tissue); Wide-spread expression (τ < 0.85); Weak expression (estimates for i-th tissue < 10 tissues); Specific expression (τ > 0.85 for only a specific i-th tissue of interest).

**3. SRTdb’s Shannon entropy measure:**

To identify tissue/cancer/cell type-specific transcripts, Shi et al., 2022 calculate a specificity score (St) computed for j-th transcript, to find tissue specific transcripts. The St is calculated as the logarithm of the total number of tissue/cancer/cell types (N) minus the Shannon entropy of its expression across these types. If a transcript's largest expression ratio in a specific tissue/cancer/cell type is more than two times greater than the second largest ratio and its St > 1, it is defined as specific to that tissue/cancer/cell type (Shi et al., 2022).

**4. HPA’s Tissue Specificity (TS) score:**

A cutoff value of nTPM is used as a limit of detection across tissues as reported in (Pontén, Jirström, & Uhlen, 2008). In the context of ^i-th^ tissue and j-th gene expression, the categories can be explained as:

1. Enriched genes – nTPM (normalized TPM expression values) > 1 in the i-th tissue while being at least four times higher than in any other tissue or type (max(nTPMij) > 4 * max(nTPMik), where k ≠ i).
2. Group enriched genes – Show similar patterns across a group of related tissues, including the i-th tissue, with nTPM > 1 and at least four times higher than in any other tissue within the group.
3. Enhanced genes - Elevated expression with nTPM > 1 in a subset of tissues, including the i-th tissue, and at least four times higher than the mean expression across other tissues in the subset (max(nTPMij) > 4 * mean(nTPMik), where k represents tissues within the subset).
4. Low specificity genes - nTPM greater >= 1 in the i-th tissue but not notably higher than in others.
5. Not detected genes - nTPM values < 1 in all tissues, including the i-th tissue.

In terms of distribution, they are defined as follows:

1. "Detected in single" genes – Specifically in the i-th tissue (max(nTPMij) > 1),
2. "Detected in some" genes - expressed in > 1 tissue and < 1/3^rd^ of tissues,
3. "Detected in many" genes - >= 1/3^rd^ of tissues,
4. "Detected in all" genes are expressed in i_all_,
5. "Not detected" genes have nTPM < 1 (i_all_).

According to the classification system, if RNA tissue specificity is labelled as "Tissue enriched" and RNA tissue distribution is marked as either "Detected in some" or "Detected in single," it is regarded as tissue specific to compare with TransTEx (Pontén, Jirström, & Uhlen, 2008).

**Supplementary Tables**

Supplementary Table 1: Number of samples based on RIN (RNA integrity number) and overall samples in GTEx v8 for isoform level mRNA expression data. RIN > 6 and RIN >=7 are the recommended thresholds. The tissues with ~~striked~~ style are not considered in the analysis of tissue specificity due to small sample sizes.

| **Tissue** | **Lower RIN value** | **Upper RIN value** | **Samples with RIN >= 7** | **Samples with RIN**  **>6** | **Total samples** |
| --- | --- | --- | --- | --- | --- |
| Adipose_Tissue | 5.5 | 10 | 572 | 1055 | 1204 |
| Adrenal_Gland | 5.5 | 10 | 188 | 229 | 258 |
| ~~Bladder~~ | 5.5 | 9 | 12 | 12 | 21 |
| Blood | 5.5 | 10 | 874 | 920 | 929 |
| Blood_Vessel | 3.2 | 10 | 795 | 1184 | 1335 |
| Brain | 5 | 10 | 1216 | 2224 | 2642 |
| Breast | 5.5 | 9.9 | 183 | 364 | 459 |
| ~~Cervix_Uteri~~ | 5.5 | 9.2 | 7 | 12 | 19 |
| Colon | 5.5 | 9.5 | 408 | 639 | 779 |
| Esophagus | 5.4 | 10 | 995 | 1272 | 1445 |
| ~~Fallopian_Tube~~ | 5.6 | 8.5 | 5 | 7 | 9 |
| Heart | 5.1 | 9.6 | 501 | 750 | 861 |
| ~~Kidney~~ | 5.5 | 8.8 | 13 | 46 | 89 |
| Liver | 5.5 | 9.8 | 77 | 176 | 226 |
| Lung | 5.1 | 9.7 | 324 | 505 | 578 |
| Muscle | 5.5 | 9.9 | 745 | 794 | 803 |
| Nerve | 5.4 | 9.5 | 275 | 529 | 619 |
| Ovary | 5.5 | 9 | 114 | 159 | 180 |
| Pancreas | 5.2 | 9.4 | 165 | 291 | 328 |
| Pituitary | 5.5 | 9.5 | 108 | 244 | 283 |
| Prostate | 5.5 | 8.7 | 75 | 174 | 245 |
| Salivary_Gland | 5.5 | 9.5 | 149 | 161 | 162 |
| Skin | 4.6 | 10 | 1361 | 1709 | 1809 |
| Small_Intestine | 5.5 | 8.8 | 124 | 173 | 187 |
| Spleen | 5.5 | 9.7 | 113 | 190 | 241 |
| Stomach | 5.5 | 9.5 | 167 | 298 | 359 |
| Testis | 5.5 | 9.3 | 102 | 285 | 361 |
| Thyroid | 5.5 | 9.7 | 251 | 551 | 653 |
| Uterus | 5.5 | 9.6 | 95 | 126 | 142 |
| Vagina | 5.5 | 9.5 | 99 | 135 | 156 |
| **Total samples** | **--** | **--** | **10113** | **15214** | **17382** |

Supplementary Table 2: Number of samples based on RIN (RNA integrity number) and overall samples in GTEx v8 for isoform level mRNA expression data. RIN > 6 and RIN >=7 are the recommended thresholds. The tissues with ~~striked~~ style are not considered in the analysis of tissue specificity due to small sample sizes.

| Tissue | L-IP_i_ | R-IPi  range (0.75, 0.9) | No. of transcripts P_ij_ < L-IP_i_ | No. of transcripts P_ij_ ≥ R-IP_i_ | No. of transcripts L-IP_i_ < P_ij_ ≤R- IP_i_ |
| --- | --- | --- | --- | --- | --- |
| Adipose Tissue | 0.17 | 0.78 | 101118 (98653) | 41260 (14544) | 2244 (2193) |
| Adrenal Gland | 0.15 | 0.9 | 105821 (103292) | 34158 (13604) | 2371 (2320) |
| Blood | 0.22 | 0.75 | 121025 (118186) | 18630 (8552) | 3433 (3356) |
| Blood Vessel | 0.19 | 0.76 | 102325 (99830) | 41823 (14520) | 2723 (2661) |
| Brain | 0.2 | 0.76 | 106736 (104197) | 36089 (15029) | 3437 (3365) |
| Breast | 0.16 | 0.75 | 95793 (93420) | 45328 (15355) | 2491 (2433) |
| Colon | 0.2 | 0.89 | 103577 (101067) | 32300 (13111) | 2224 (2173) |
| Esophagus | 0.2 | 0.75 | 103097 (100591) | 39302 (14077) | 3276 (3209) |
| Heart | 0.24 | 0.76 | 121390 (118578) | 30791 (12851) | 3317 (3245) |
| Liver | 0.14 | 0.75 | 114160 (111497) | 31914 (12741) | 3159 (3090) |
| Lung | 0.17 | 0.78 | 93914 (91570) | 47219 (15969) | 3205 (3132) |
| Muscle | 0.17 | 0.83 | 118337 (115595) | 25727 (11308) | 3125 (3061) |
| Nerve | 0.19 | 0.81 | 95344 (92963) | 48697 (16262) | 1952 (1902) |
| Ovary | 0.19 | 0.9 | 100236 (97761) | 38263 (14205) | 2846 (2777) |
| Pancreas | 0.16 | 0.88 | 119551 (116752) | 26079 (12013) | 3286 (3221) |
| Pituitary | 0.2 | 0.82 | 99267 (96840) | 47598 (16848) | 2556 (2494) |
| Prostate | 0.17 | 0.89 | 96294 (93931) | 39803 (15106) | 2145 (2094) |
| Salivary Gland | 0.19 | 0.79 | 101851 (99359) | 43255 (15404) | 3313 (3241) |
| Skin | 0.21 | 0.82 | 100750 (98292) | 34333 (12894) | 1948 (1909) |
| Small Intestine | 0.2 | 0.78 | 101524 (99043) | 43417 (15558) | 2766 (2706) |
| Spleen | 0.2 | 0.89 | 103979 (101456) | 40446 (15201) | 2779 (2716) |
| Stomach | 0.18 | 0.87 | 105793 (103266) | 30508 (12771) | 3148 (3084) |
| Testis | 0.2 | 0.76 | 88963 (86739) | 66362 (23156) | 2405 (2352) |
| Thyroid | 0.2 | 0.77 | 97164 (94774) | 50591 (16680) | 2241 (2189) |
| Uterus | 0.17 | 0.89 | 95884 (93499) | 39845 (14643) | 1770 (1723) |
| Vagina | 0.18 | 0.76 | 97422 (95019) | 45999 (15677) | 3337 (3265) |
| Union of all transcripts |  |  | 133242 (50319) | 91742 (27592) | 3706 (2679) |
| Intersection of all transcripts |  |  | 57757 (22275) | 11072 (1063) | 457 (410) |

Supplementary Table 3: Distribution of alternative transcripts of TSp genes across other expression groups. For genes (column 1) of transcripts (bold and highlighted) that belong to an expression group, counts (% in parentheses) of alternative transcripts of those genes that fall in other expression groups are reported in columns 3-7.

| Total number of | | Number of alternative transcripts in other expression groups | | | | |
| --- | --- | --- | --- | --- | --- | --- |
| Genes | Transcripts | Low | Null | TEn | TSp | Wide |
| 27431 | 158735 | 79191 (50%) | 30965 (20%) | 5653 (4%) | 11705 (7%) | 31221 (20%) |
| 33975 | 139512 | 48507 (35%) | 57757 (41%) | 3878 (3%) | 8413 (6%) | 20957 (15%) |
| 4566 | 37373 | 14613 (39%) | 7010 (19%) | 7436 (20%) | 3411 (9%) | 4903 (13%) |
| 10635 | 70377 | 25634 (36%) | 13532 (19%) | 3047 (4%) | 17999 (26%) | 10165 (14%) |
| 13899 | 112712 | 47347 (42%) | 19798 (18%) | 2947 (3%) | 5837 (5%) | 36783 (33%) |

Supplementary Table 4: Distribution of expression classes across transcripts not found in TransTEx-based classification.

| **Class** | **Tau-score** ∉ **TransTExDb** | **SRTdb** ∉ **TransTExDb** | **Tau-score ∉ SRTdb** |
| --- | --- | --- | --- |
| Null | 2246 | 29132 | 1565 |
| Low | 96 | 310 | 58 |
| TEn | 3063 | 2094 | 2125 |
| Wide | 225 | 1868 | 148 |
| Total | 5630 | 33404 | 3896 |

Supplementary Table 5: Distribution of gene-TransTEx expression class pairs where gene IDs are compared with HPA.

| **Class** | **HPA** ∉ **TransTExDb** | **HPA** ∉ **SRTdb** | **HPA** ∉ **Tau-score** |
| --- | --- | --- | --- |
| Null | 748 | 560 | 647 |
| Low | 1 | 0 | 1 |
| TEn | 138 | 95 | 62 |
| Wide | 1 | 1 | 5 |
| Total | 888 | 656 | 715 |

Supplementary Table 6: Other expression classes in TransTEx that the 3,446 multi-transcript mapped genes are expressed in which may contain other transcripts of the same gene.

Note: Genes are shared across the expression classes as the real comparison here is at the isoform level hence the total number of genes is summing up to more than the actual number of unique genes.

| **Condition** | **Number of genes** | | **Number of transcripts** | |
| --- | --- | --- | --- | --- |
| Null | 2836 | (96%) | 18403 | (76%) |
| Low | 307 | (10%) | 442 | (2%) |
| TEn | 792 | (27%) | 1534 | (6%) |
| Wide | 1294 | (44%) | 3877 | (16%) |
| **Total** | **2963** | | **24256** | |

Supplementary Table 7: Number of TSp transcripts and the gene associated to find the gene which has the maximum number of TSp transcripts from the analysis.

**Supplementary Table 7A:** Summarizes the number of TSp transcripts present in multiple tissues.

| **Ensembl Gene ID** | **Gene Symbol** | **Ensembl Transcript ID** | **Number of TSp transcripts** | **Number of tissues** |
| --- | --- | --- | --- | --- |
| ENSG00000125462 | *MIR9-1HG* | ENST00000310027,ENST00000357975,ENST00000368242,ENST00000368243,ENST00000400991,ENST00000441085,ENST00000452465,ENST00000464203,ENST00000465270,ENST00000465570,ENST00000469813,ENST00000471156,ENST00000484428,ENST00000486517,ENST00000489877,ENST00000495000,ENST00000497822,ENST00000497824,ENST00000608007 | 19 | 16 Brain, 3 Testis |
| ENSG00000197971 | *MBP* | ENST00000354542,ENST00000355994,ENST00000397869,ENST00000397875,ENST00000447114,ENST00000473302,ENST00000483025,ENST00000498683,ENST00000579129,ENST00000581179,ENST00000581878,ENST00000582282 | 12 | 8 Brain, 2 Muscle, 2 Nerve |
| ENSG00000067606 | *PRKCZ* | ENST00000400921,ENST00000419838,ENST00000461106,ENST00000470986,ENST00000471018,ENST00000478770,ENST00000482686,ENST00000486681,ENST00000497183,ENST00000503297 | 10 | 3 Brain, 2 Lung, 5 Testis |
| ENSG00000154556 | *SORBS2* | ENST00000393528,ENST00000421420,ENST00000425679,ENST00000437304,ENST00000445625,ENST00000464975,ENST00000470685,ENST00000476311,ENST00000478249,ENST00000493709 | 10 | 2 Blood_Vessel, 7 Heart, 1 Thyroid |
| ENSG00000087460 | *GNAS* | ENST00000371102,ENST00000462499,ENST00000472183,ENST00000480232,ENST00000482112,ENST00000490374,ENST00000491348,ENST00000493744 | 8 | 1 Pituitary, 1 Salivary_Gland, 6 Testis |
| ENSG00000105048 | *TNNT1* | ENST00000586282,ENST00000587758,ENST00000588147,ENST00000588426,ENST00000589226,ENST00000589745,ENST00000592920,ENST00000593046 | 8 | 1 Brain, 7 Muscle |
| ENSG00000144847 | *IGSF11* | ENST00000425327,ENST00000441144,ENST00000459718,ENST00000480431,ENST00000483401,ENST00000489689,ENST00000491903,ENST00000494802 | 8 | 1 Nerve, 7 Testis |
| ENSG00000171759 | *PAH* | ENST00000549111,ENST00000550978,ENST00000551114,ENST00000551988,ENST00000552251,ENST00000553106,ENST00000635477,ENST00000635528 | 8 | 7 Liver, 1 Testis |
| ENSG00000176884 | *GRIN1* | ENST00000371546,ENST00000371550,ENST00000371553,ENST00000371559,ENST00000371561,ENST00000462584,ENST00000473811,ENST00000485413 | 8 | 7 Brain, 1 Salivary_Gland |
| ENSG00000082397 | *EPB41L3* | ENST00000341928,ENST00000545076,ENST00000579271,ENST00000581292,ENST00000581387,ENST00000581833,ENST00000584055 | 7 | 1 Brain, 1 Pituitary, 5 Testis |

**Supplementary Table 7B:** Summarizes the number of TSp transcripts present in a single tissue.

| **Ensembl Gene ID** | **Gene Symbol** | **Ensembl Transcript ID** | **Number of Tsp transcripts** | **Tissue** |
| --- | --- | --- | --- | --- |
| ENSG00000187021 | *PNLIPRP1* | ENST00000358834,ENST00000470678,ENST00000471549,ENST00000482159,ENST00000482833,ENST00000484402,ENST00000497792,ENST00000510125,ENST00000525157,ENST00000525820,ENST00000526223,ENST00000527980,ENST00000528052,ENST00000529584,ENST00000530319,ENST00000530626,ENST00000531825,ENST00000534513,ENST00000534537 | 19 | Pancreas |
| ENSG00000124678 | *TCP11* | ENST00000311875,ENST00000373974,ENST00000394696,ENST00000412155,ENST00000427376,ENST00000444780,ENST00000445851,ENST00000455706,ENST00000469514,ENST00000479418,ENST00000486638,ENST00000502480,ENST00000503908,ENST00000504758,ENST00000505335,ENST00000505911,ENST00000512012 | 17 | Testis |
| ENSG00000095794 | *CREM* | ENST00000342105,ENST00000361599,ENST00000374721,ENST00000395887,ENST00000427847,ENST00000461968,ENST00000463314,ENST00000464475,ENST00000466251,ENST00000469949,ENST00000482646,ENST00000487132,ENST00000494479,ENST00000495301,ENST00000495960,ENST00000496626 | 16 | Testis |
| ENSG00000131095 | *GFAP* | ENST00000435360,ENST00000585728,ENST00000586125,ENST00000586127,ENST00000586793,ENST00000588037,ENST00000588316,ENST00000588735,ENST00000589701,ENST00000590922,ENST00000591327,ENST00000591719,ENST00000638281,ENST00000638618,ENST00000639277,ENST00000639921 | 16 | Brain |
| ENSG00000042832 | *TG* | ENST00000518097,ENST00000518108,ENST00000518505,ENST00000519178,ENST00000519294,ENST00000520089,ENST00000520197,ENST00000520769,ENST00000521107,ENST00000522797,ENST00000522809,ENST00000522996,ENST00000523756,ENST00000523901,ENST00000524151 | 15 | Thyroid |
| ENSG00000118194 | *TNNT2* | ENST00000367317,ENST00000367318,ENST00000367320,ENST00000412633,ENST00000438742,ENST00000445079,ENST00000455702,ENST00000466570,ENST00000472177,ENST00000475686,ENST00000476888,ENST00000477035,ENST00000494095,ENST00000509001,ENST00000515042 | 15 | Heart |
| ENSG00000066813 | *ACSM2B* | ENST00000329697,ENST00000562026,ENST00000564849,ENST00000564855,ENST00000565232,ENST00000565322,ENST00000566998,ENST00000567001,ENST00000567288,ENST00000568098,ENST00000569131,ENST00000569163,ENST00000569344,ENST00000569364 | 14 | Liver |
| ENSG00000163631 | *ALB* | ENST00000415165,ENST00000476441,ENST00000484992,ENST00000495173,ENST00000503124,ENST00000504043,ENST00000505649,ENST00000507673,ENST00000508932,ENST00000509063,ENST00000510166,ENST00000514786,ENST00000515133 | 13 | Liver |
| ENSG00000183747 | *ACSM2A* | ENST00000396104,ENST00000570698,ENST00000571204,ENST00000572843,ENST00000572921,ENST00000573854,ENST00000574251,ENST00000574692,ENST00000575690,ENST00000576101,ENST00000576119,ENST00000576361 | 12 | Liver |
| ENSG00000257017 | *HP* | ENST00000357763,ENST00000561927,ENST00000562488,ENST00000562526,ENST00000564499,ENST00000565574,ENST00000565807,ENST00000566821,ENST00000567185,ENST00000567612,ENST00000570083,ENST00000576168 | 12 | Liver |

**Supplementary Table 7C:** Frequency of the number of genes which map to the number of transcripts for instance, 1835 genes map to 2 transcripts each.

| **TSp transcripts mapped to a single gene** | **Number of genes** |
| --- | --- |
| 2 | 1672 |
| 3 | 707 |
| 4 | 334 |
| 5 | 173 |
| 6 | 91 |
| 7 | 53 |
| 8 | 21 |
| 9 | 22 |
| 10 | 11 |
| 11 | 9 |
| 12 | 2 |
| 13 | 1 |
| 14 | 1 |
| 15 | 2 |
| 16 | 2 |
| 17 | 1 |
| 19 | 1 |
| Total number of genes | 3103 |

**Supplementary Table 9:** Brain regions tissue specific transcripts across 10 different brain regions.

**Supplementary Table 9A:** Brain regions are compared with the different expression groups in TransTEx.

|  | LowTrans | NullTrans | TenhTrans | TspTrans | WideTrans | Total |
| --- | --- | --- | --- | --- | --- | --- |
| Amygdala | 1 | 0 | 0 | 1 | 0 | 2 |
| Cerebellar_Hemisphere | 14 | 0 | 0 | 2 | 2 | 18 |
| Cerebellum | 93 | 6 | 3 | 4 | 36 | 142 |
| Cortex | 1 | 0 | 0 | 0 | 0 | 1 |
| Hippocampus | 2 | 0 | 0 | 0 | 0 | 2 |
| Hypothalamus | 4 | 0 | 1 | 1 | 3 | 9 |
| Nucleus_accumbens  (basal_ganglia) | 17 | 0 | 3 | 5 | 1 | 26 |
| Putamen  (basal_ganglia) | 0 | 0 | 0 | 1 | 0 | 1 |
| Spinal_cord  (cervical_c-1) | 111 | 9 | 21 | 22 | 48 | 211 |
| Substantia_nigra | 3 | 0 | 1 | 0 | 1 | 5 |
| Total | 246 | 15 | 29 | 36 | 91 | 417 |

**Supplementary Table 9B:** Brain regions are compared with the other tissues the brain-region specific transcripts are enriched in. None of them are enriched in Brian in the analysis.

|  | Esophagus | Heart | Liver | Lung | Muscle | Nerve | Ovary | Pituitary | Spleen | Testis | Total |
| --- | --- | --- | --- | --- | --- | --- | --- | --- | --- | --- | --- |
| Amygdala | 0 | 0 | 0 | 1 | 0 | 0 | 0 | 0 | 0 | 0 | 1 |
| Cerebellar_Hemisphere | 0 | 0 | 0 | 0 | 1 | 0 | 0 | 0 | 0 | 1 | 2 |
| Cerebellum | 0 | 0 | 1 | 0 | 0 | 1 | 0 | 0 | 0 | 2 | 4 |
| Hypothalamus | 0 | 0 | 0 | 0 | 0 | 0 | 0 | 0 | 0 | 1 | 1 |
| Nucleus_accumbens  (basal_ganglia) | 0 | 1 | 0 | 0 | 0 | 0 | 0 | 1 | 0 | 3 | 5 |
| Putamen  (basal_ganglia) | 0 | 0 | 0 | 0 | 1 | 0 | 0 | 0 | 0 | 0 | 1 |
| Spinal_cord  (cervical_c-1) | 1 | 0 | 3 | 0 | 0 | 4 | 1 | 1 | 1 | 11 | 22 |
| Total | 1 | 1 | 4 | 1 | 2 | 5 | 1 | 2 | 1 | 18 | 36 |

**Supplementary Table 10:** Alternative tissue sites among the TSp genes of Brain, Liver, Testis, Muscle and Heart.

| TSp Site | TSp + Cell Type  Marker Genes | Number of Genes | | Number of Multi Transcripts | |
| --- | --- | --- | --- | --- | --- |
|  |  | Single Transcript | Multi Transcript | Site | 2nd Alternate Site (transcripts) |
| Brain | 251 | 190 | 61 | 176 | Testis (22) |
| Liver | 393 | 194 | 199 | 615 | Testis (54) |
| Testis | 5663 | 3295 | 2368 | 6865 | Brain (102) |
| Muscle | 262 | 146 | 116 | 239 | Testis (73) |
| Heart | 84 | 47 | 37 | 87 | Testis (18) |

**Supplementary Figures**

Supplementary Figure 1: Tau-score calculation cutoff decided by calculating inflection point cutoff.


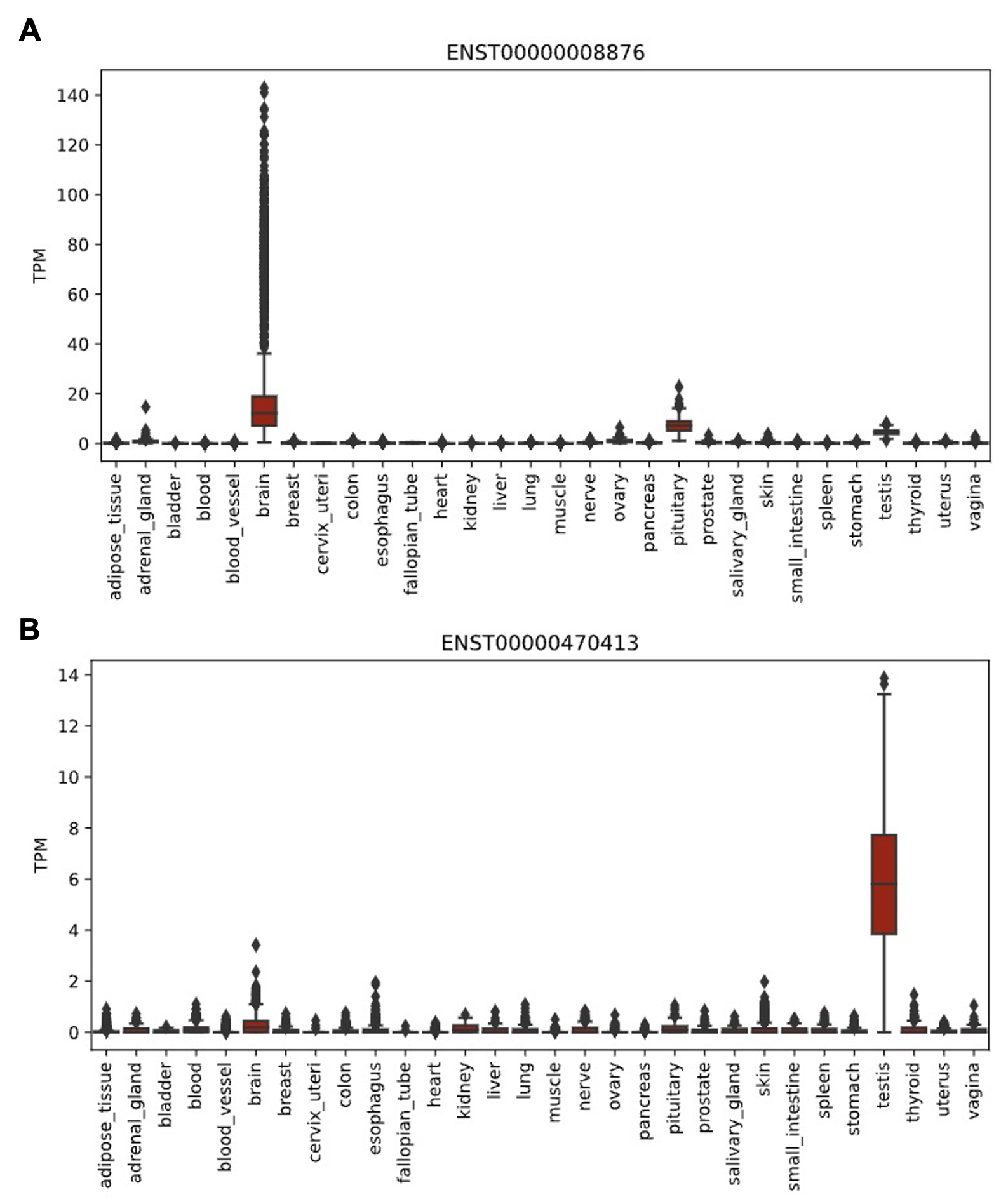


**Supplementary Figure 2:** (A) ENST00000008876 maps to MAPK8IP2 gene and is brain-specific according to Tau-score, but clearly it shows enhanced in brain, pituitary and testis as identified by TransTEx. (B) ENST00000470413 maps to CENPS-CORT genes and is brain-specific according to SRTdb but is classified as testis-specific according to TransTEx.


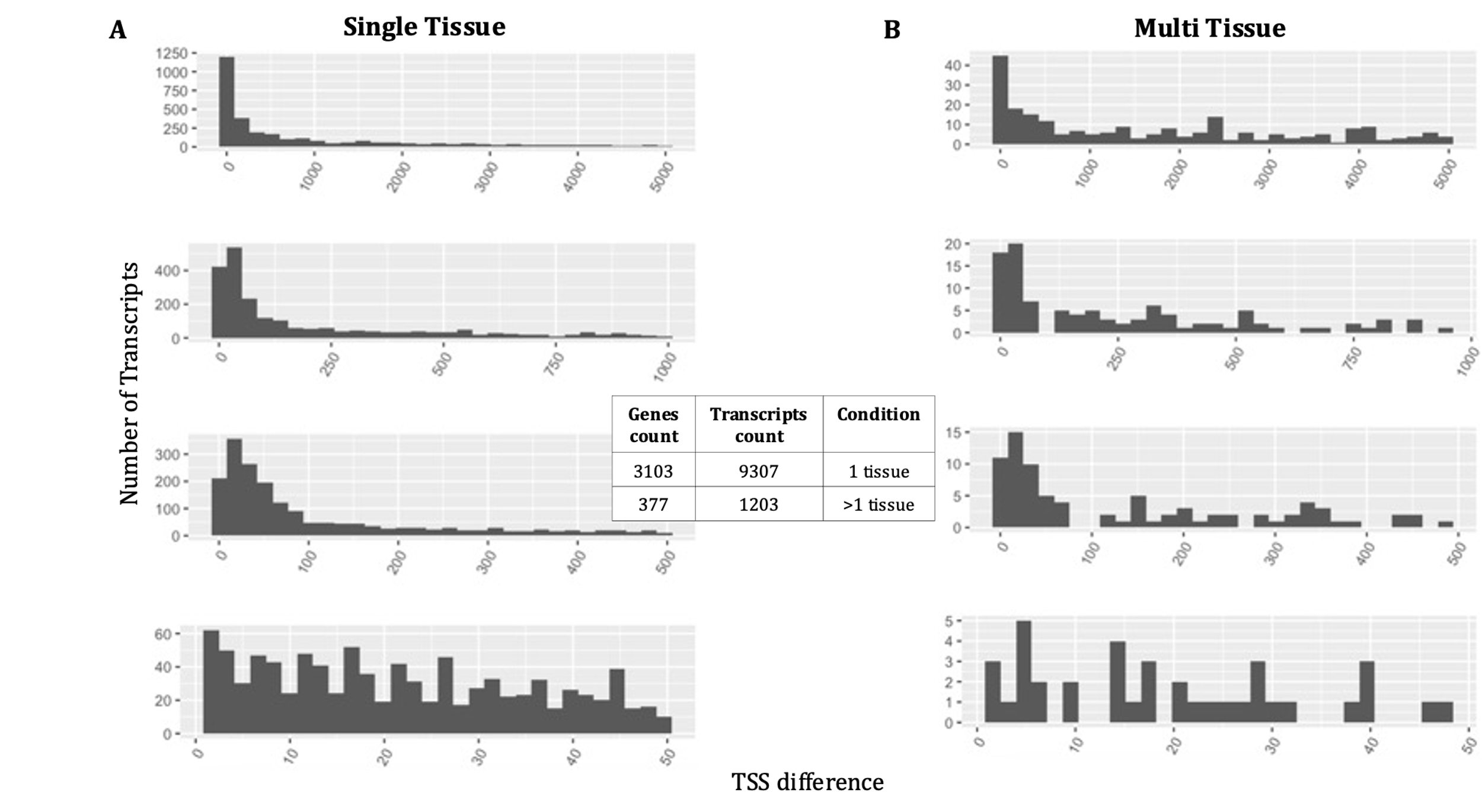


Supplementary Figure 3: Single tissue and multi tissue groups TSS difference to distinguish between the transcripts that belong to the 2 groups.


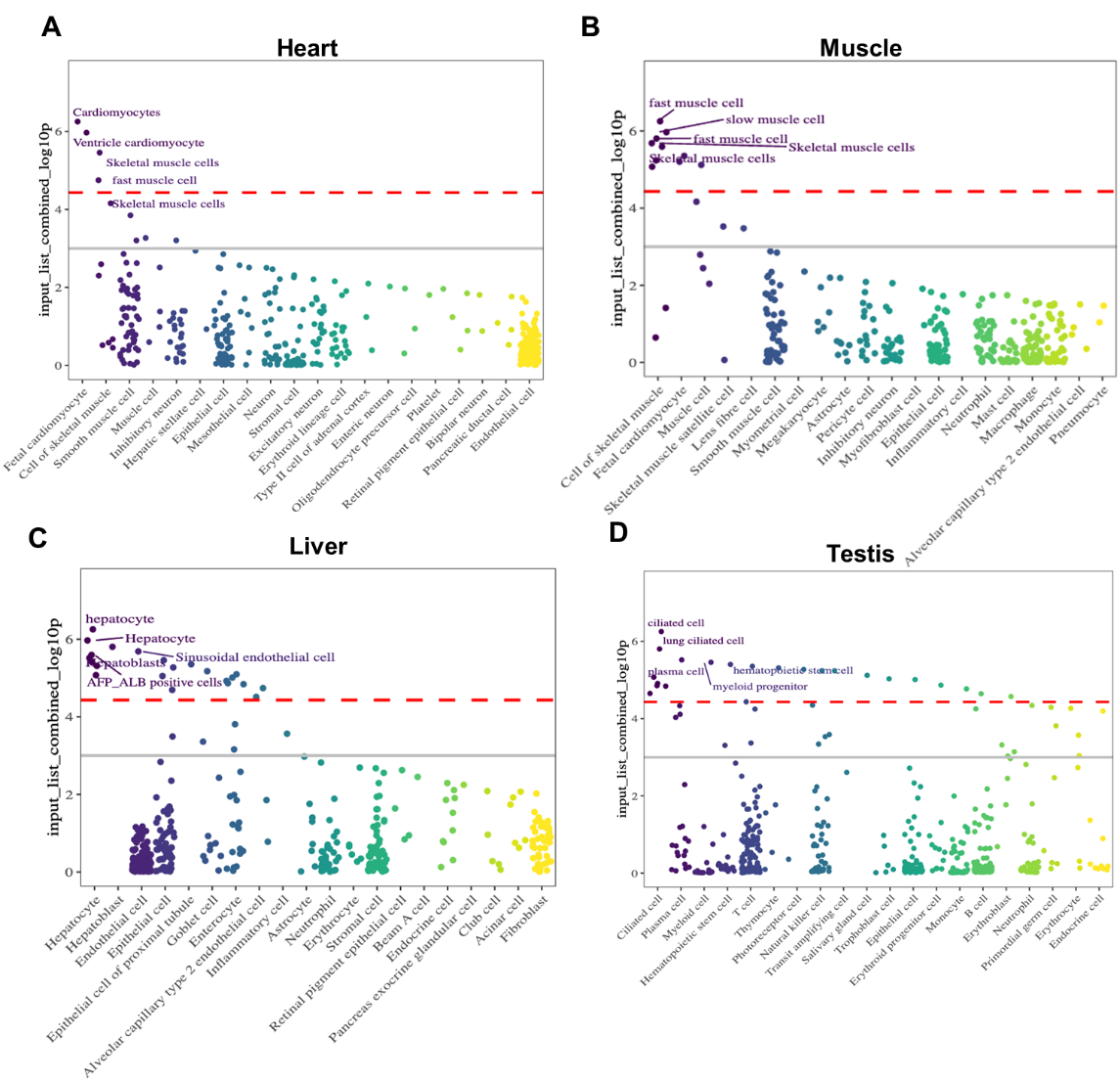


Supplementary Figure 4: Top 20 Cell types enriched from WebCSEA in the genes mapped to TSp sites. (A) Heart TSP genes; (B) Muscle TSp genes; (C) Liver TSp genes and (D) Testis TSp genes.

References

Duffy, Á., Verbanck, M., Dobbyn, A., Won, H.-H., Rein, J. L., Forrest, I. S., Nadkarni, G., Rocheleau, G., & Do, R. (2020). Tissue-specific genetic features inform prediction of drug side effects in clinical trials. *Science Advances*, *6*(37), eabb6242. https://doi.org/doi:10.1126/sciadv.abb6242

Kryuchkova-Mostacci, N., & Robinson-Rechavi, M. (2017). A benchmark of gene expression tissue-specificity metrics. *Briefings in bioinformatics*, *18*(2), 205-214.

Moreno, P., Fexova, S., George, N., Manning, J. R., Miao, Z., Mohammed, S., Muñoz-Pomer, A., Fullgrabe, A., Bi, Y., & Bush, N. (2022). Expression Atlas update: gene and protein expression in multiple species. *Nucleic acids research*, *50*(D1), D129-D140.

Pontén, F., Jirström, K., & Uhlen, M. (2008). The Human Protein Atlas—a tool for pathology. *The Journal of Pathology: A Journal of the Pathological Society of Great Britain and Ireland*, *216*(4), 387-393.

Pundir, S., Ji, Y., Shilpi, A., & Davuluri, R. V. (2021). Stratified Subsampling Based p-values for Hypothesis Tests in Genomics Research. *Stat Appl*, *19*(1), 1-13.

Shi, Q., Liu, T., Hu, W., Chen, Z., He, X., & Li, S. (2022). SRTdb: an omnibus for human tissue and cancer-specific RNA transcripts. *Biomarker Research*, *10*(1), 1-11.
